# 

**Published:** 1863-03

**Authors:** 


New Series, Vol. 6, No. 3. Whole Series, Vol. 20, No. 3.

Box 1305

### THE CHICAGO MEDICAL JOURNAL.

DANIEL BRAINARD, M. D.,
J. ADAMS ALLEN, M. D.,
Editors and Proprietors.

MARCH, 1863.

CHICAGO:
J. C. W. BAILEY, BOOK AND JOB PRINTER, 130 CLARK STREET.
1863.

